# P-1331. Efficacy of Levofloxacin for Levofloxacin-Susceptible, Ciprofloxacin-Nonsusceptible Enterobacterales

**DOI:** 10.1093/ofid/ofaf695.1519

**Published:** 2026-01-11

**Authors:** Madison Salam, Meghan N Jeffres

**Affiliations:** UCHealth University of Colorado Hospital, Denver, Colorado; University of Colorado Anschutz Medical Campus, Aurora, CO

## Abstract

**Background:**

Fluoroquinolones (FQ) are critical Gram-negative agents due to their oral availability and stability against beta-lactamases such as ESBL and AmpC. Resistant organisms frequently co-harbor FQ resistance genes. There are copious resistance mechanisms and no widely available genotypic analysis, yet *in vitro* studies suggest a higher resistance risk for discordant susceptibilities. Discordance is documented in a subset of Enterobacterales isolates; there is a paucity of literature describing FQ efficacy in this population. We aim to evaluate microbiologic and clinical success of patients receiving levofloxacin LVX-Susceptible (LVX-S), Ciprofloxacin-nonsusceptible (CIP-NS) Enterobacterales.

**Table 1.**
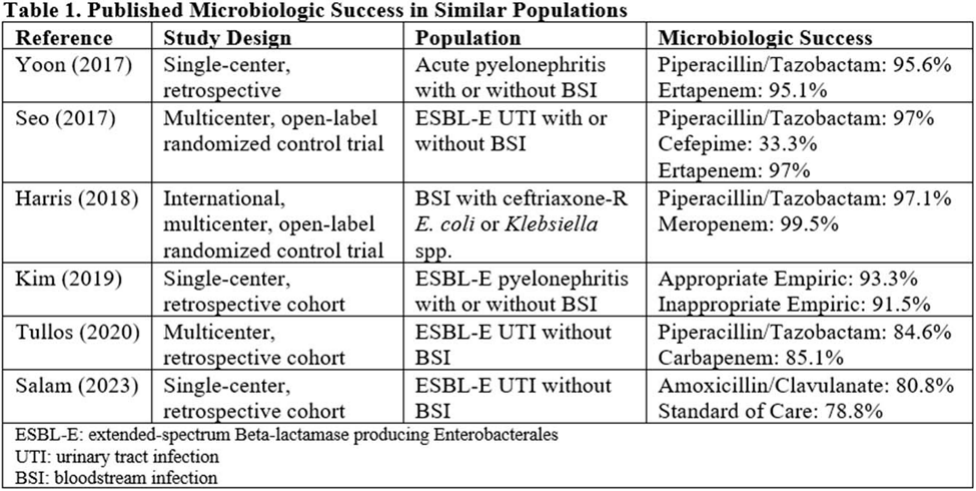
Published Microbiologic Success in Similar Populations

**Methods:**

This is a single-arm, retrospective, descriptive analysis of patients aged 18-89 treated for LVX-S, CIP-NS Enterobacterales who received LVX. Susceptibility testing was performed via Phoenix BMD with the exception of *Serratia* (Kirby Bauer). Primary outcome was 90d microbiologic failure, defined as growth of the same organism within 90d of index culture. Secondary outcomes were growth of LVX-R isolates, 90d treatment failure defined as retreatment regardless of cultures, receipt of salvage therapy, and 90d all-cause mortality.

**Results:**

Preliminary reporting identified 73 patients; 48 were included. Median age was 60.0 and patients were predominantly female (66.7%). Most common organism was *K. pneumoniae* (41.7%). The majority (85.4%) were ceftriaxone-R. Microbiologic failure occurred in 14 (29.2%) of patients. LVX-R isolates grew in 4 patients. Treatment failure occurred in 11 (22.9%) patients. 90d all-cause mortality was 14.6%. Post hoc analysis of risk factors for composite failure was not significant, including receipt of high-dose LVX (OR = 0.2; 95% CI: 0.1-1.1).

**Conclusion:**

In this cohort of LVX-treated patients with LVX-S, CIP-NS isolates showed 90d microbiologic failure in 29.2% of patients, which is higher than published literature. Lower FQ dose may be a risk factor for failure. This study has significant limitations including its design and small sample size. To our knowledge, this is the first study of clinical outcomes for FQ-treated patients with discordant FQ sensitivities. Results should be confirmed with larger analyses.

**Disclosures:**

All Authors: No reported disclosures

